# Comparison of testing of collimator and beam alignment, focal spot size with slit camera, and tube current consistency using computed radiography and conventional screen‐film systems

**DOI:** 10.1002/acm2.12600

**Published:** 2019-05-16

**Authors:** Tipvimol Meechai, Khaisang Chousangsuntorn, Wiwat Owasirikul, Manus Mongkolsuk, Woranut Iampa

**Affiliations:** ^1^ Department of Radiological Technology, Faculty of Medical Technology Mahidol University Thailand; ^2^ Faculty of Radiological Technology Rangsit University Thailand

**Keywords:** physical performance test, quality control of x‐ray tube, computed radiography, digital imaging, pixel value

## Abstract

Conversion to a filmless technique of physical performance testing is becoming a topic of much interest to researchers. We assessed the use of a computed radiography (CR) system with postprocessing software as an alternative tool for performing the three physical performance tests of an x‐ray tube. Collimator and beam alignment, focal spot size, and milliampere second (mAs) linearity, were performed using a CR system. Results were then compared with those obtained from a conventional screen‐film (SF) system. The distances of collimator misalignment measured by the SF system were decreased while peak tube voltage (kVp) was increased (mAs was fixed), whereas those measured by CR were independent of exposure level. The degrees of beam collimator misalignment measured by the CR system were not different from those measured by the SF system. The differences in focal spot dimensions measured by SF and CR systems were less than 4% for large and small focal spot size in both width and length. The mAs linearity evaluated by the SF system agreed with those evaluated by the dose measurement at 50 kVp and 4 mAs, as well as 55 kVp and 3.2 mAs, while the mAs linearity test using the CR system agreed with those using the dose measurement method for all exposure levels. In summary, a CR system could be utilized to assess the three physical performance tests of a single x‐ray tube, but required more time than an SF system. Medical physicists with image processing skills were needed to perform the analyses.

## 1 INTRODUCTION

Digital imaging and advanced computer technology are becoming widely utilized in hospitals since they allow image manipulation and long‐term image storage.^1^ A cassette‐based photostimulable storage phosphor and plate reader system, known as a “computed radiography” (CR) system, has replaced analog screen‐film (SF) while allowing use of existing x‐ray infrastructure. CR images can be transferred to picture archiving and communication systems (PACS) and displayed to radiologists for diagnosis. Even though CR images can be adjusted after exposure, there are several factors which affect image quality. Radiological technologists are the key people who are responsible for delivering good quality images, while minimizing radiation exposure to patients. Physical performance tests of the technical parameters generated from an x‐ray tube are very important for achieving good quality CR images.^2^

In this study, we evaluated a filmless method of testing the physical performance of an x‐ray tube, including collimator and beam alignment, focal spot size, and milliampere second (mAs) linearity, using a CR system instead of a screen‐film (SF) system. Beam alignment testing was performed to evaluate shift of the center of the x‐ray beam from the vertical line. Collimator alignment testing was performed to verify whether a light field had the same alignment as an x‐ray field. The mAs linearity test was done to assess the consistency of tube current at the same mAs. Measurement of focal spot size was performed initially during the acceptance test and then occasionally as part of image quality control.^3^ Independent of SF system, radiochromic film (Gafchromic) was developed for dose measurement in radiation therapy and other dosimetry applications in radiology.^4^ One new model, XR‐QA Gafchromic film, was designed for kilovoltage dosimetry with increased sensitivity to detect lower doses and optimize energy dependence.^5^ However, the film’s response is energy dependent, with the response being significantly reduced at lower energies such as 15 and 28 keV (corresponding to 28 and 40 kVp, respectively). The Gafchromic XR film’s sensitivity is significantly higher for radiation beams with energies in the range of 44–71 keV (80–140 kVp), but this may not be the range most suitable for the physical performance testing of x‐ray tubes in this study.

We found few publications that reported the use of filmless modalities for measurement of focal spot sizes. In 2003, Rong et al.^3^ reported on the utilization of a CR imaging plate and flat panel detector for measurement of focal spot size, and the comparison of these results with those measured by an SF system. In 2011, Russo et al.^6^ proposed a new method based on a coded aperture mask for measurement of focal spot size, and then compared the results with those measured by a slit camera using a flat panel detector. Conversion to a filmless alternative technique of physical performance testing of x‐ray tubes is a topic of increasing interest to researchers. We hypothesized that CR could be used for testing the physical performance of x‐ray tubes. Thus, we assessed the use of a CR system (CR imaging plate and CR reader) with postprocessing software by performing the three physical performance tests of an x‐ray tube, that is, collimator and beam alignment, focal spot size, and mAs linearity. We report here the comparison between output measurements using a CR system and those obtained from an SF system (screen‐film cassette and film processor) which was the standard tool recommended by the National Council on Radiation Protection & Measurements (NCRP) Report No. 99.^7^

## 2 MATERIALS AND METHODS

Three physical performance tests of x‐ray tubes (collimator and beam alignments, focal spot size, and mAs linearity) were studied using a CR system. All of these tests were performed and interpreted based on the standards of NCRP Report No. 99.^7^ Prior to performing the assessments, the x‐ray machine, CR (imaging plate and reader) and SF (screen‐film cassette and film processer) systems, and related equipment to be used (e.g., dosimeter) had been quality checked and calibrated. Siemens x‐ray machine (model Quantum Medical Imaging) of the Golden Jubilee Medical Center, Salaya, Nakhon Pathom, was checked for accuracy and precision of tube voltage and exposure time, and reproducibility of exposure [all according to NCRP No. 99 recommendations].^7^

Quality control tests of the SF system were based on NCRP Report No. 99,^7^ while those of the CR system were based on American Association of Physicists in Medicine Report No. 93.^8^ These were performed prior to use in this study. The SF system consisted of 8″ × 10″ Kodak X‐OMAT cassettes and regular screen, and a Kodak X‐OMAT multiloader 7000 processor with clinical setting at 35ºC temperature. The CR system consisted of 8”×10” imaging plates (FUJI‐FILM IP cassette type C) corresponding to 100.0 μm in pixel size and a CR imaging reader (FUJI‐FILM FCR XG5000). CR images were processed in fixed EDR mode (S = 200) using a test/linearity menu, with a linear gradation look‐up table and no frequency processing. Brightness levels of the view box, which was used for film‐visual interpretation, were adjusted to the National Electrical Manufacturers Association (NEMA)’s standard range (700–2000 cd/m^2^). We investigated the effects of differing exposure levels on all three physical performance parameters being tested. Various exposure levels, which were generated in each study by varying peak tube voltage (kVp) and/or mAs, were measured by a calibrated, Radcal model 9095 dosimeter (Radcal Corporation, Monrovia, CA, USA).

### 2.1 Beam and collimator alignment test

**Figure 1:**
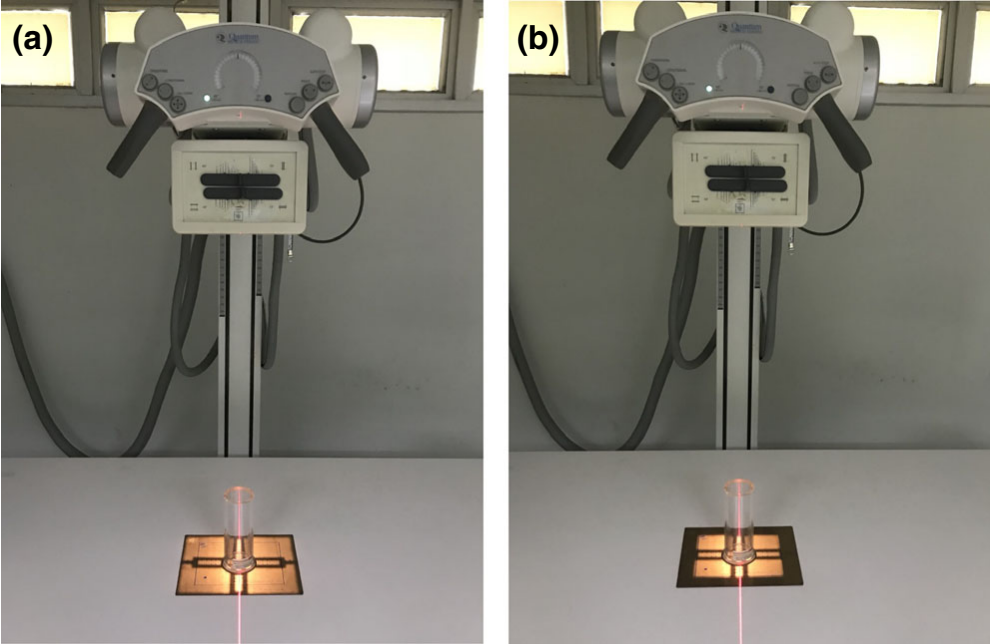
Double exposures were performed in the collimator and beam alignments tests: (a) first exposure, light field covered the outer borders of the test tool plate to make a background density on the image; (b) second exposure, light field was collimated to fit the inner rectangular outline of the test tool

First, the RMI beam alignment test tool (Model 161B, Radiation Measurements Inc, Middleton, WI 53567, USA), which had two lead balls located on the top and bottom of the test tool, was put on the center of the collimator alignment plate. Both collimator and beam alignment test tools were placed on the table at the center of the light field. We inserted an 8″ × 10″ screen‐film cassette into the Bucky tray. Source to image distance (SID) was set to 100 cm. Secondly, we collimated the light field to cover all of the outer border of the collimator alignment plate [Fig. 1(a)]. The screen‐film cassette was exposed to create the background optical density (OD). Then, we adjusted the collimator shutter until the edges of the light field covered the inner border (rectangular outline) of the collimator test tool [Fig. 1(b)]. To investigate the effects of exposure on collimator and beam alignments, screen‐films were exposed at various exposure levels (fixed tube current of 100 mA; varied kVp and mAs) that could retrieve ODs ranging from 1.0 to 3.0, meeting NEMA requirements.^9^ The exposed films were processed using a film processor. Finally, we repeated the same experiments but used 8″ × 10″ CR imaging plates instead of the screen‐film cassettes. The CR imaging plates were read by the CR imaging reader. The digital images were sent to the postprocessing software for analysis.

Beam alignment was evaluated by measuring the distance between the centers of the two lead balls, then calculating the angle of beam misalignment (in degrees) using Equation (1):

(1) $$\theta =\tan^{-1}\left[\frac{\mathrm{rb}}{a\left(a+b+x\right)}\right]$$

where, a = height of beam alignment test tool; b = distance from focal spot to the top of beam alignment test tool; r = distance between two lead balls; x = distance from table top to film.

For the analyses of the films, visual interpretation was performed using a view box and a ruler. The distances of collimator misalignment in both short and long axes, and the distance between two separated lead balls as they appeared on the film, were measured by a single observer (T.M.) to eliminate the variability in image interpretation which may occur with multiple observers.^10^

**Figure 2:**
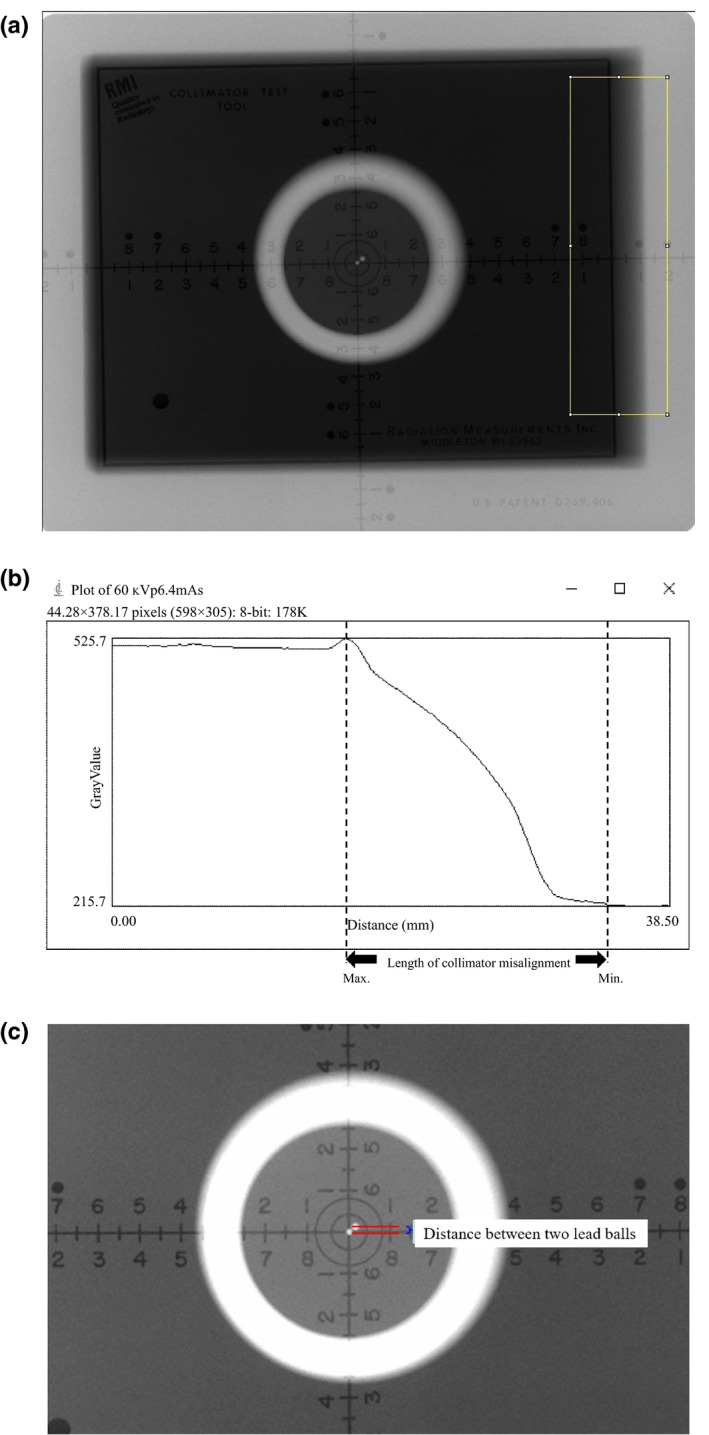
Interpretation of the collimator and beam alignments using the computed radiography image: (a) drawing regions of interest to cover 90% of the area; (b) plotting the gray value profiles; (c) measurement of the distance of beam misalignment (distance between the centers of two lead balls), which was used for calculating the angle of beam misalignment

For the analyses of the CR images, the images in a DICOM format were exported to the ImageJ1.36b+ software program. For the measurement of collimator misalignment, rectangular regions of interest (ROIs) were drawn to cover 90% of the border area of an individual edge [Fig. 2(a)]. Then, the profiles of the gray values measured from the selected ROIs were plotted. Distances between the highest (represented by a lead line at the border of the test tool) and lowest (represented by the outermost region of the x‐ray field) points of gray values were measured [Fig. 2(b)]. Those distances represented the misalignment between x‐ray and light fields.^11^ Misalignments of collimators were measured in both short and long axes. For the assessment of angles of beam misalignment, we measured the distance between the centers of the two separated lead balls which appeared on the screen using the ImageJ measuring tool [Fig. 2(c)] and then calculated the angle of beam misalignment using Equation (1) from above.

According to the NCRP Report No. 99,^7^ the distance of collimator misalignment must not exceed 2% of SID and the angle of beam misalignment must not exceed 3°. The results obtained from both films and CR images were assessed based on the same criteria.

### 2.2 Measurement of focal spot size using a slit camera

First, the height adjustable stand (Gammex model RMI‐07‐642) was placed on the table. The compatible alignment tool was placed on top of the adjustable stand for setting the proper alignment of the stand in relation to the reference axis.^10^ Then, we removed the alignment tool and put the slit camera (Nuclear Associates, Carle Place, NY) with a slit width of 10 μm in the alignment tool insert so that the reference axis intersected the middle of the slit camera. An 8″ × 10″ screen‐film cassette was placed in the base of the adjustable stand. To obtain the selected nominal focal spot designation (F), the alignment tool to film distance and SID distance were adjusted to obtain the correct enlargement factor (E) based on Table 1. The small nominal focal spot was 0.6 mm and the large nominal focal spot was 1.2 mm. Then we replaced the alignment tool with a slit camera. The slit camera was placed parallel and perpendicular to the anode–cathode axis for measuring width and length, respectively, of both the small and large focal spots. To investigate the effect of exposure on the focal spot sizes, we used x‐rays at various exposure levels (fixed tube current of 100 mA, varied kVp and mAs) which could produce an OD of 1.4^9^ on the screen‐film cassette. The exposed films were processed using the film processor. For assessment of focal spot size with the CR system, we performed the same procedures but used CR imaging plates (8″ × 10″) instead of screen‐film cassettes.

**Table 1:** Nominal focal spot designation and enlargement factor (NEMA standard).

Nominal focal spot designation (F)	Enlargement factor (E)
F < 0.4	E > 3
0.4 ≤ F ≤ 1.0	E > 2
1.0 < F	E > 1

The enlargement factor of the slit camera image itself is the slit‐image distance divided by the source‐slit distance) = M − 1.

For the analyses of the films, visual interpretation was performed by a single observer (T.M.) using a view box, ruler and magnifying glass which had a built‐in graticule of 0.01 mm divisions and 7X magnification.^9^ The dimensions of the focal spot were determined from a pair of slit images. The extent of the discernible image was measured over each focal spot slit image normal to the length of the slit at half of its length. It was possible to perceive a contrast step of approximately 5%. The relationship between the imaged focal spot size and actual focal spot size is shown in Equation (2):

(2) $$a_{W,L}=\left(I_{W,L}-sM\right)/M-1$$

where, a = actual focal spot dimension (length & width); I = image dimension (length & width); s = slit width; M = standard geometrical magnification factor (SID divided by the source to slit distance).

The dimensions of the maximum focal spot were calculated by dividing the dimensions of the focal spot image by the factor E.

**Figure 3:**
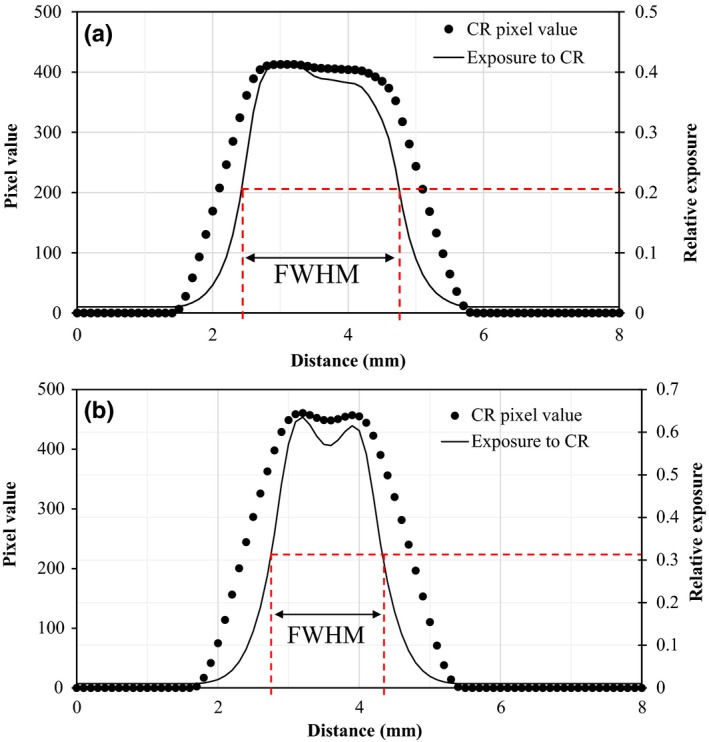
FWHM measurement of small focal spot size using a computed radiography image: (a) slit profile of slit image perpendicular to the anode‐cathode axis and full width at the half‐maximum (FWHM) measured at half of the peak relative exposure to calculate the length of the focal spot; (b) slit profile of slit image parallel to the anode‐cathode axis and FWHM measured at half of the highest peak relative exposure to calculate the width of the focal spot

For the analyses of the CR images, the images with 10‐bit depth in a DICOM format were exported to the ImageJ1.36b+ software program. A digital profile was measured on the original 10‐bit CR image by using the ImageJ software program, and then 8‐bit profile data were shown in the ImageJ window. The profile data were exported to determine a full width at the half maximum (FWHM). The dimension of slit image in CR was calculated from FWHM of the focal spot image. It should be mentioned that the NEMA standard does not provide criteria for visually determining the width of slit image. The NEMA standard states that “the extent of the discernible image shall be measured over each focal spot slit image normal to the length of the slit at half of its length.”^9^ However, no specific instructions are provided on how to determine the boundaries of “the extent of the discernible image” It has been our practice to measure the dimension of a slit image by visually estimating the FWHM of that image. To measure the dimension of a slit image from a digital profile, we applied the same criterion to ensure consistency in the evaluation. A slit image perpendicular to the anode–cathode axis was drawn ROI to cover 90% of the area and the FWHM of slit profile was measured to calculate the length of the focal spot. A slit image parallel to the anode–cathode axis was drawn ROI to cover 90% of the area and the FWHM of slit profile was measured to calculate the width of the focal spot. In addition, for the radiation profile recorded by the film, the half maximum of optical density and that of relative exposure occur at the same point. However, for the radiation profile acquired using CR, the half‐maximal pixel value and half‐maximal relative exposure do not occur at the same point. Therefore, in this study, the FWHM using CR measurement was determined at the half maximum of relative exposure based on the imaging plate characteristic function.^12^ FWHM was determined coordinately at half of the peak relative exposure of a single peak profile [Fig. 3(a)], while the FWHM was determined at half of the highest relative exposure as the maximum value of the twin peak profile [Fig. 3(b)]. The focal spot size was calculated from the FWHM using Equation (3)^3^:

(3) $$FSS=\left(\text{FWHM}-0.01\times M\right)/\left(M-1\right),$$

where FSS represents focal spot size in mm; M is the magnification factor; and the factor 0.01 is the physical slit width in mm. The image magnification factor was determined from the ratio of the size of the slit camera image and the physical size of the slit camera. The magnification factors ranged from 3.07 to 3.24.^3^

The maximum focal spot dimension tolerance limits were based on NEMA standards.^9^ Focal spot sizes from both films and CR images were evaluated based on the same criteria.

### 2.3 mAs linearity test

Prior to performing the mAs linearity test by imaging an aluminum step wedge, a linearity test was perform by the standard method using dose measurement.^7^, ^13^ At each exposure level (with varied kVp and/or mAs), the exposure values were measured by a calibrated Radcal model 9095 dosimeter at the same kVp and mAs with 100 and 200 mA stations. At each station, the exposure values were measured five times, and then the coefficient of variation or linearity variance was calculated to evaluate the mAs linearity. The acceptable coefficient of variation or linearity variance should be within 0.1 or 10%, respectively.^7^, ^13^

**Figure 4:**
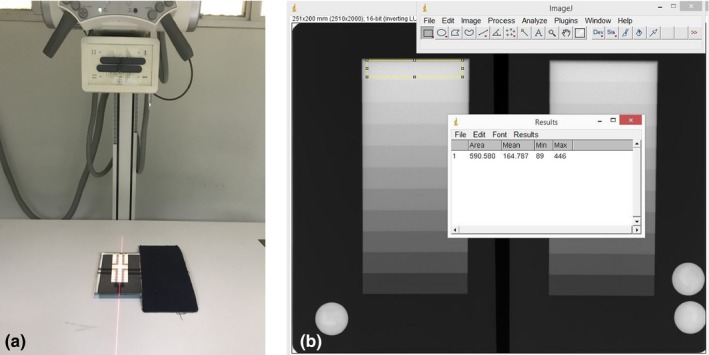
mAs linearity test: (a) experimental setup; (b) measurement of pixel value of each step by drawing regions of interest to cover 90% of the area of each step without overlapping the adjacent steps

First, an 8″ × 10″ screen‐film cassette was split into two portions. An 11‐step aluminum wedge (RMI serial no.117‐1908) was placed on the left side of the screen‐film. We covered the unexposed (right) side with a lead plate. Center of the x‐ray beam was pointed at the middle step of the 11‐step aluminum wedge, then the x‐ray field was collimated to just cover the left side of the film [Fig. 4(a)]. SID was 100 cm and tube current was 100 mA. The x‐ray film was exposed to produce the step wedge image. Then the same procedure was performed on the other side, but with the tube current changed to 200 mA. To investigate the effect of exposure on mAs linearity, films were exposed at various exposure levels (with varied kVp and/or mAs). The exposed films were processed by the film processor. Finally, we performed the same procedures, but used CR imaging plates (8″ × 10″) instead of the screen‐films.

For the analyses of the films, we measured the ODs of all 11 steps using a calibrated densitometer (X‐rite 301, Incorporated USA). For the analyses of the CR images, we measured the pixel value (PV) of all 11 steps using the ImageJ1.36b+ software program by drawing rectangular ROIs which covered 90% of the area of each step without overlapping adjacent steps [Fig. 4(b)]. Both ODs and PVs measured on the same step of the images from the different tube currents were checked for consistency. We assumed that any mA and time that produced the same mAs should have produced the same ODs on the films as well as the same PVs on the CR images. By imaging an aluminum step wedge, the acceptable variation in the optical densities should be within ±0.1 for the SF system and of the pixel values should be within 20% of each other for the CR system.^13^, ^14^

## 3 RESULTS

Based on NCRP Report No. 99,^7^ distances of collimator misalignment must not exceed 2 cm (2% of SID) on both short and long axes, and the angle of beam misalignment must not exceed 3 degrees. Table 2 presents comparisons of the distances (in cm) of collimator misalignment and the angles (in degrees) of beam misalignment measured by SF and CR systems at various exposure levels. The results of collimator and beam alignment tests obtained from both the SF and CR systems passed the NCRP criteria. The distances of collimator misalignment in short and long axes measured by the SF system were 1.18 ± 0.32 cm (min = 0.70 cm, max = 1.55 cm) and 1.28 ± 0.21 cm (min = 0.96 cm, max = 1.49 cm), while those measured by the CR system were 1.86 ± 0.03 cm (min = 1.83 cm, max = 1.90 cm) and 1.78 ± 0.03 cm (min = 1.72, max = 1.82 cm), respectively. The means of angles of beam misalignment were 0.3° on every exposure level for both the SF and CR systems. The distances of collimator misalignment (Table 2) for the CR system were greater than those for the SF system in both short (57.1% greater, that is, mean of 1.86 vs 1.18) and long (39.9% greater, that is, mean of 1.78 vs 1.28) axes, while no differences in the angles of beam misalignment were found for either the short or long axes (i.e., mean of 0.3).

**Table 2:** Distances of collimator misalignment and angles of beam misalignment measured at various exposure levels compared between those of the screen‐film (SF) and computed radiography (CR) systems.

Tube voltage (kVp)	mAs	Exposure (mR)	Short axis (cm)			Long axis (cm)			Angle (°)		
			SF	CR	d (%)^a^	SF	CR	d (%)^a^	SF	CR	d (%)^a^
55	5.0	7.96	1.53	1.83	19.6	1.48	1.78	20.3	0.3	0.3	0.0
	6.4	11.13	1.45	1.89	30.3	1.49	1.81	21.5	0.3	0.3	0.0
	7.5	15.27	1.55	1.82	17.4	1.47	1.75	19.0	0.3	0.3	0.0
60	5.0	9.57	1.38	1.90	37.7	1.32	1.72	30.3	0.3	0.3	0.0
	6.4	13.46	1.30	1.86	43.1	1.40	1.81	29.3	0.3	0.3	0.0
	7.5	18.61	1.13	1.90	68.1	1.33	1.80	35.3	0.3	0.3	0.0
70	5.0	13.21	0.84	1.84	119	0.96	1.75	82.3	0.3	0.3	0.0
	6.4	18.82	0.70	1.80	157	0.98	1.82	85.7	0.3	0.3	0.0
	7.5	26.13	0.76	1.87	146	1.02	1.78	74.5	0.3	0.3	0.0
Mean			1.18	1.86	57.1	1.28	1.78	39.9	0.3	0.3	0.0
SD			0.32	0.03	52.24	0.21	0.03	26.51	0.0	0.0	0.0

a Percent difference from SF, %.

Based on the NEMA standards,^9^ the width and length of the focal spot must not exceed 0.90 mm and 1.30 mm for F = 0.60 mm (small focal spot), nor 1.70 mm and 2.40 mm for F = 1.20 mm (large focal spot) respectively. For a small focal spot size (F = 0.60 mm), the width and length measured by the SF system were 0.80 mm and 1.20 mm, while those measured by the CR system ranged 0.78–0.83 and 1.16–1.23, respectively (Table 3). For large focal spot size (F = 1.20 mm), the width and length measured by the SF system were 1.30 mm and 1.80 mm, while the width measured by the CR system were 1.34 and the length ranged from 1.78 to 1.83 (Table 4). These results indicated that the measured small and large focal spot sizes obtained from both SF and CR systems passed the NEMA standard’s criteria. The differences in focal spot dimensions measured by the SF system and the CR system (shown in Tables 3 and 4) were less than 4%.

**Table 3:** Slit camera measurement: small focal spot sizes (nominal focal spot size: 0.60 mm) measured at various exposure levels, compared between those of the screen‐film (SF) and computed radiography (CR) systems.

Tube voltage (kVp)	mAs	Exposure (mR)	Width (mm)		d (%)^a^	Length (mm)		d (%)^a^
			SF	CR		SF	CR	
60	2.4	8.30	0.80	0.83	3.75	1.20	1.18	−1.67
60	3.0	12.27	0.80	0.78	−2.50	1.20	1.18	−1.67
70	1.8	6.17	0.80	0.78	−2.50	1.20	1.16	−3.33
90	1.2	7.27	0.80	0.83	3.75	1.20	1.23	2.50
CV (%)			0.0%	3.1%		0.0%	2.2%	

a Percent difference from SF, %.

**Table 4:** Slit camera measurement: large focal spot sizes (nominal focal spot size: 1.20 mm) measured at various exposure levels, compared between those of the screen‐film (SF) and computed radiography (CR) systems.

Tube voltage (kVp)	mAs	Exposure (mR)	Width (mm)		d (%)^a^	Length (mm)		d (%)^a^
			SF	CR		SF	CR	
75	2.4	12.94	1.30	1.34	2.50	1.80	1.78	−1.11
75	3.2	19.47	1.30	1.34	2.50	1.80	1.83	1.67
80	2.0	10.61	1.30	1.34	2.50	1.80	1.83	1.67
CV			0.0%	0.0%		0.0%	1.4%	

a Percent difference from SF, %.

Based on the mAs linearity test by the standard method using dose measurement,^7^, ^13^ the acceptable coefficient of variation should be within 0.1. In this study, the coefficient of variation in mAs linearity ranged from −0.01 to 0.09 as shown in Table 5. The results from the dose measurement method indicated that the mAs linearity at each exposure level was acceptable. By imaging an aluminum step wedge, the acceptable variation in the optical densities should be within ±0.1 for the SF system. We found that the results of linearity testing using the SF system agreed with those using the dose measurement for the exposure levels at 50 kVp and 4 mAs, as well as at 55 kVp and 3.2 mAs (Table 5). For the CR system, the acceptable variation in pixel values should be within 20%. The results of the linearity testing using the CR system (Table 6) agreed with those using the dose measurements for all exposure levels.

**Table 5:** mAs linearity test using the dose measurement and the screen‐film system: optical densities measured at various exposure levels compared between those of 100 and 200 mA.

Step no.	50 kVp, 4 mAs				55 kVp, 2 mAs				55 kVp, 2.4 mAs				55 kVp, 3.2 mAs				62 kVp, 3.2 mAs				73 kVp, 2.5 mAs			
	200 mA	100 mA	d^a^	$\left[\overset{¯}{x}\right]$	200 mA	100 mA	d^a^	$\left[\overset{¯}{x}\right]$	200 mA	100 mA	d^a^	$\left[\overset{¯}{x}\right]$	200 mA	100 mA	d^a^	$\left[\overset{¯}{x}\right]$	200 mA	100 mA	d^a^	$\left[\overset{¯}{x}\right]$	200 mA	100 mA	d*	$\left[\overset{¯}{x}\right]$
1	0.33	0.34	0.01	0.34	0.30	0.31	0.01	0.31	0.34	0.35	0.01	0.35	0.46	0.47	0.01	0.47	0.43	0.49	0.06	0.46	0.45	0.53	0.08	0.49
2	0.33	0.34	0.01	0.34	0.31	0.31	0.00	0.31	0.36	0.40	0.04	0.38	0.48	0.48	0.00	0.48	0.46	0.55	0.09	0.51	0.50	0.60	0.10	0.55
3	0.35	0.34	−0.01	0.35	0.32	0.34	0.02	0.33	0.43	0.46	0.03	0.45	0.57	0.58	0.01	0.58	0.60	0.72	0.12	0.66	0.64	0.75	0.11	0.70
4	0.43	0.42	−0.01	0.43	0.37	0.39	0.02	0.38	0.52	0.60	0.08	0.56	0.80	0.83	0.03	0.82	0.81	0.94	0.13	0.88	0.81	0.99	0.18	0.90
5	0.63	0.64	0.01	0.64	0.46	0.52	0.06	0.49	0.69	0.80	0.11	0.75	1.13	1.17	0.04	1.15	1.11	1.28	0.17	1.20	1.08	1.29	0.21	1.19
6	0.97	0.97	0.00	0.97	0.58	0.68	0.10	0.63	0.95	1.09	0.14	1.02	1.59	1.62	0.03	1.61	1.49	1.66	0.17	1.58	1.44	1.69	0.25	1.57
7	1.49	1.51	0.02	1.50	0.81	0.96	0.15	0.89	1.36	1.55	0.19	1.46	2.02	2.05	0.03	2.04	1.89	2.02	0.13	1.96	1.89	2.15	0.26	2.02
8	2.01	2.02	0.01	2.02	1.18	1.39	0.21	1.29	1.92	2.14	0.22	2.03	2.37	2.39	0.02	2.38	2.26	2.35	0.09	2.31	2.39	2.57	0.18	2.48
9	2.43	2.43	0.00	2.43	1.75	1.99	0.24	1.87	2.47	2.66	0.19	2.57	2.63	2.64	0.01	2.64	2.56	2.6	0.04	2.58	2.74	2.90	0.16	2.82
10	2.71	2.72	0.01	2.72	2.40	2.60	0.20	2.50	2.86	2.97	0.11	2.92	2.79	2.83	0.04	2.81	2.77	2.8	0.03	2.79	2.98	3.07	0.09	3.03
11	2.86	2.87	0.01	2.87	2.89	2.97	0.08	2.93	3.08	3.12	0.04	3.10	2.89	2.92	0.03	2.91	2.90	2.88	−0.02	2.89	3.12	3.15	0.03	3.14
Expo‐ sure (mR)	3.05	3.68	CV = 0.09	3.37	1.53	1.50	CV = ‐0.01	1.52	1.97	2.11	CV = 0.01	2.04	2.56	3.08	CV = 0.09	2.82	3.54	3.96	CV = 0.06	3.75	3.43	3.75	CV = 0.02	3.59

$\left[\overset{¯}{x}\right]$, mean; CV, coefficient of variation in mAs linearity resulting from the dose measurement.

a Difference in optical density from 200 mA.

**Table 6:** mAs linearity test using the computed radiography system: pixel values measured at various exposure levels compared between those of 100 and 200 mA.

**Step no.**	50 kVp, 4 mAs				55 kVp, 2 mAs				55 kVp, 2.4 mAs				55 kVp, 3.2 mAs				62 kVp, 3.2 mAs				73 kVp, 2.5 mAs			
	200 mA	100 mA	d^a^ (%)	$\left[\overset{¯}{x}\right]$	200 A	100 mA	d^a^(%)	$\left[\overset{¯}{x}\right]$	200 mA	100 mA	d^a^ (%)	$\left[\overset{¯}{x}\right]$	200 mA	100 mA	d^a^ (%)	$\left[\overset{¯}{x}\right]$	200 mA	100 mA	d^a^ (%)	$\left[\overset{¯}{x}\right]$	200 mA	100 mA	d^a^ (%)	$\left[\overset{¯}{x}\right]$
1	39.7	38.7	−2.5	39.2	42.5	47.5	11.8	45.0	54.9	63.9	16.4	59.4	62.6	65.7	4.9	64.2	65.6	68.8	4.7	67.2	71.2	77.7	9.1	74.5
2	42.6	42.0	−1.4	42.3	47.6	54.9	15.3	51.2	60.9	70.1	15.1	65.5	69.2	73.1	5.6	71.1	72.5	76.6	5.4	74.5	79.6	86.4	8.5	83.0
3	55.2	55.1	−0.2	55.1	59.2	67.2	13.5	63.2	72.7	82.3	13.2	77.5	81.5	85.4	4.8	83.5	85.4	89.5	4.6	87.4	90.5	97.4	7.6	93.9
4	69.7	69.2	−0.7	69.5	73.0	81.1	11.1	77.0	85.5	95.8	12.0	90.7	95.0	99.1	4.3	97.0	99.5	103.8	4.1	101.6	102.0	109.6	7.5	105.8
5	85.3	84.8	−0.6	85.0	87.8	95.6	8.9	91.7	99.5	109.9	10.5	104.7	109.4	113.5	3.8	111.5	114.6	118.9	3.6	116.8	114.5	122.3	6.8	118.4
6	101.6	100.8	−0.8	101.2	103.5	110.8	7.1	107.1	113.7	124.5	9.5	119.1	124.3	128.3	3.2	126.3	130.2	134.4	3.1	132.3	127.4	135.1	6.0	131.3
7	118.5	117.6	−0.8	118.1	119.4	126.7	6.1	123.1	128.3	139.4	8.7	133.8	139.6	143.5	2.8	141.6	146.2	150.4	2.8	148.3	140.2	148.8	6.1	144.5
8	135.8	135.0	−0.6	135.4	136.0	143.0	5.1	139.5	143.4	154.9	8.0	149.2	155.5	159.5	2.6	157.5	162.9	167.1	2.5	165.0	153.7	162.5	5.7	158.1
9	154.3	153.2	−0.7	153.7	154.1	160.2	4.0	157.2	160.2	171.7	7.2	166.0	172.9	176.4	2.1	174.6	181.1	184.8	2.0	183.0	168.3	177.4	5.4	172.9
10	174.4	173.2	−0.7	173.8	173.6	179.1	3.2	176.4	178.0	190.0	6.7	184.0	191.9	195.1	1.7	193.5	201.0	204.4	1.7	202.7	184.1	193.7	5.2	188.9
11	197.1	195.7	−0.7	196.4	195.6	200.5	2.5	198.1	197.7	210.0	6.2	203.8	212.7	215.4	1.3	214.1	222.8	225.7	1.3	224.3	201.6	211.5	4.9	206.6

$\left[\overset{¯}{x}\right]$, mean.

a Percent difference from 200 mA, %.

## 4 DISCUSSION

In collimator and beam alignment tests, the distances of collimator misalignment measured by the CR system were greater than those measured by the SF system in both the long (39.9%) and short (57.1%) axes (Table 2). We found that greater differences were observed at greater exposure levels. The collimator misalignment measures obtained from the SF system tended to be less at the higher exposure levels particularly when we increased kVp and fixed mAs, while those obtained from the CR system were largely unchanged in both the short and long axes. Evaluation of collimator alignment using the SF system was qualitative (visual interpretation), and when using the CR system was quantitative (measurement from the plotted pixel number profiles). The CR system was an exposure‐independent method of collimator alignment test of x‐ray tubes. Using the CR system with the postprocessing software reduced uncertainty from human interpretation and response differences in films at various exposure levels that might have led to the underestimation of the collimator misalignment distance. We also found that beam misalignment results with the CR system were not different from those with the SF system. High contrast between two separated lead balls which appeared on the center of the films was easy to differentiate from the background [Fig. 2(c)].

In focal spot size measurement using a slit camera, we found that discrepancies between focal spot sizes with the CR system and those with the SF system were less than 4%. The results show that variations in the focal spot sizes measured using the CR system among various exposure levels ranged from 0% to 3.1%. We also found that the focal spot sizes were not affected by variation in exposure level in either CR or SF system (Tables 3 and 4). The results indicated that measurement of focal spot size can be performed precisely across a wide range of exposure levels with either the CR or SF system, in agreement with the investigation of Rong et al.^3^ However, the high exposure level caused the signal saturation of imaging plates^12^ and may result in wider FWHM. Therefore, the appropriate exposure level for focal spot sizes measurement using CR system should be investigated.

Regarding mAs linearity testing, the results of SF system agreed with those of dose measurement at 50 kVp and 4 mAs, as well as 55 kVp and 3.2 mAs, while the results of CR system agreed with those of dose measurement for all exposure levels. Due to a wide exposure dynamic range of CR over SF systems, the results demonstrated that the mAs linearity testing using CR system can be utilized on various exposure levels. In contrast, the mAs linearity testing using SF system may require appropriate exposure techniques for accurate evaluation.

**Figure 5:**
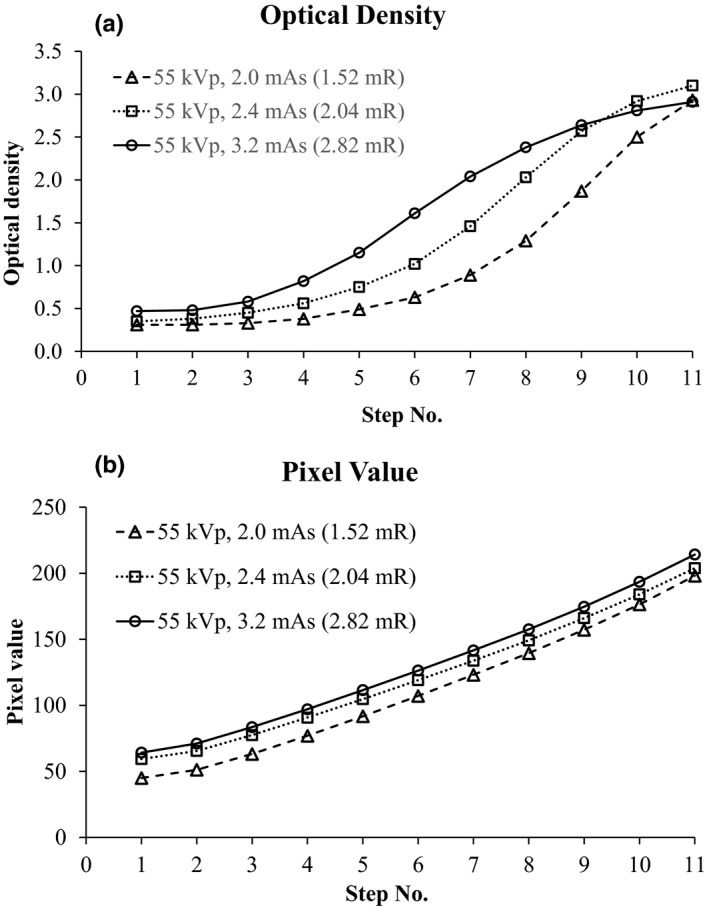
Comparisons of optical densities (a) and pixel values (b) at various exposure levels (fixed 55 kVp, varied mAs)

**Figure 6:**
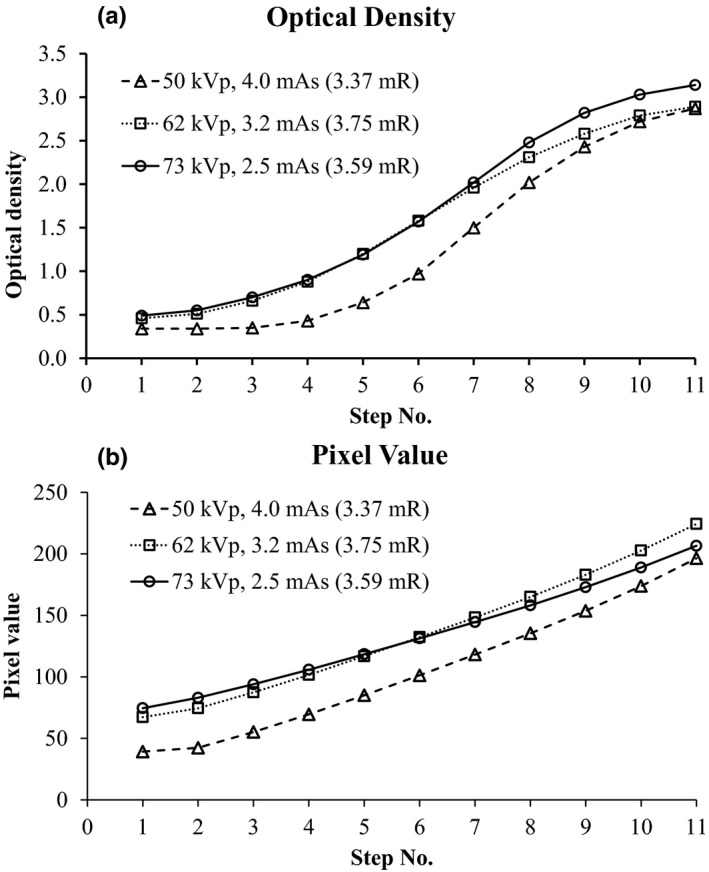
Comparisons of optical densities (a) and pixel values (b) at various exposure levels (varied kVp, varied mAs)

**Figure 7:**
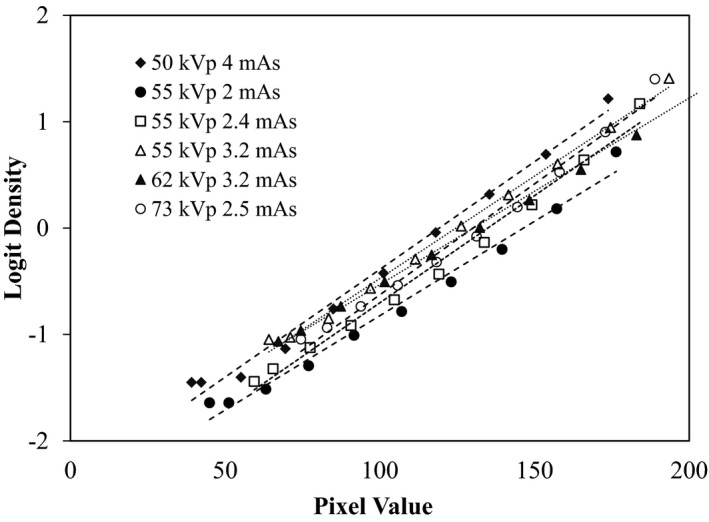
Plots of pixel values and logit densities at various exposure levels with linear approximations

In mAs linearity testing, ODs increased with aluminum step number in a sigmoid manner, while PVs increased with a more linear relationship with step number. Although there were different patterns of responses to x‐ray exposure between the film and CR receptors, the response trends of PVs and ODs were the same at every exposure level. Figure 5 presents the comparisons of the responses of ODs [Fig. 5(a)] and PVs [Fig. 5(b)] at the fixed 55 kVp and varied mAs (2.0, 2.4, and 3.2 mAs). Figure 6 presents the comparisons of ODs [Fig. 6(a)] and PVs [Fig. 6(b)] on both varied kVp (50, 62, and 73 kVp) and varied mAs (4.0, 3.2, and 2.5 mAs). We found that the responses of PVs were dependent on the exposure level [Figs. 5(b) and 6(b)] and consistent with the responses of ODs to the same exposure level [Figs. 5(a) and 6(a)]. In addition, we assessed the relationship between the ODs and the PVs with Pearson’s correlation. A *P* < 0.05 was considered statistically significant for between‐group correlation. We found a strong correlation [high Pearson correlation coefficient (r)] between the means of PVs and ODs at both 100 mA (*r* = 0.964, *P* < 0.001) and 200 mA (*r* = 0.965, *P* < 0.001). The logistic functions of ODs (logit density) were plotted against the PVs to assess their relationship.^15^ Regression analysis was applied to determine a linear correlation coefficient. We found strong linear relationships [high coefficient of determination (*r* ^2^)] between the PVs and logit density at both 100 mA (*r* ^2^ ranged from 0.981 to 0.993, *P* < 0.001) and 200 mA (*r* ^2^ ranged from 0.984 to 0.997, *P* < 0.001). Figure 7 demonstrates the fitted‐linear relationships between the means of PVs and the logit of mean density at various exposure levels.

The duration of preparing experiments and data analyses (per a single exposure level) of the three physical performance tests, that is, collimator and beam alignment, focal spot size, and mAs linearity, was 60 and 80 min for the SF and CR systems, respectively (Table 7). Using the CR system took 33% longer than using the SF system due to the processing of the digital image with software compared with direct visual interpretation.

**Table 7:** Estimated time (per single exposure level) with screen‐film (SF) and computed radiography (CR) systems.

Measurements	Time (min)	
	SF	CR
Beam and collimator	15	20
Focal spot size	30	40
mAs linearity	15	20
Total	60	80

## 5 CONCLUSION

Based on the evaluations of pixel values, a CR system could be used as a filmless alternative tool for assessing collimator and beam alignments, focal spot size (using a slit camera), and mAs linearity of our x‐ray machine. Medical physicists who had digital image processing skills were needed for the analysis. Further study should include the use of CR systems with x‐ray machines from other manufacturers.

## CONFLICT OF INTEREST

No conflicts of interest.
